# Correction: Wang et al. LC–MS/MS Analysis of Choline Compounds in Japanese-Cultivated Vegetables and Fruits. *Foods* 2020, *9*, 1029

**DOI:** 10.3390/foods11152272

**Published:** 2022-07-29

**Authors:** Wenhao Wang, Shohei Yamaguchi, Masahiro Koyama, Su Tian, Aya Ino, Koji Miyatake, Kozo Nakamura

**Affiliations:** 1 Department of Science and Technology, Graduate School of Medicine, Science and Technology, Shinshu University, 8304, Minamiminowa, Nagano 399-4598, Japan; 20hs502e@shinshu-u.ac.jp (W.W.); 19hs505d@shinshu-u.ac.jp (S.Y.); 2 Wellnas Co., Ltd., Toranomon Masters Building 6F, 1-12-14, Toranomon, Minato-ku, Tokyo 105-0001, Japan; mkoyama32@wellnas.biz; 3 Department of Nutrition and Food Hygiene, School of Public Health, Hebei Medical University, Shijiazhuang 050017, China; sutianjia@yahoo.co.jp; 4 Kochi Agricultural Research Center, 1100 Hataeda, Nankoku, Kochi 783-0023, Japan; aya_ino@ken4.pref.kochi.lg.jp; 5 Institute of Vegetable and Floriculture Science, NARO, 360 Kusawa, Ano-cho, Tsu, Mie 514-2392, Japan; miya0424@affrc.go.jp; 6 Institute of Agriculture, Academic Assembly, Shinshu University, 8304, Minamiminowa, Nagano 399-4598, Japan

The authors wish to make a correction to the published version of their paper [1].

**Conflicts of Interest:** The authors declare no conflict of interest.

should be

**Conflicts of Interest:** M.K. is a shareholder-CEO in Wellnas Co., Ltd. K.N. is an inventor of pending (WO2015147251 and WO2018070545) and awarded (Japanese Patent No. 6192167 and Indonesia Patent No. P000063624) patents regarding this work and a shareholder in Wellnas Co., Ltd.

We apologize for this error. The change does not affect the scientific results. The published version will be updated on the article webpage, with a reference to this correction notice.
